# Job Replacement Anxiety and Approach-Avoidance Job Crafting: The Roles of Regulatory Focus and AI Literacy

**DOI:** 10.3390/bs16091519

**Published:** 2026-08-28

**Authors:** Chuanhao Fan, Han Wang

**Affiliations:** School of Business, Hohai University, Nanjing 211100, China

**Keywords:** job replacement anxiety, AI literacy, regulatory focus, job crafting, conservation of resources theory, AI anxiety

## Abstract

As artificial intelligence (AI) becomes increasingly embedded in organizational processes, concerns about job replacement by AI have become increasingly widespread and gradually influenced work behaviors. However, existing research has not yet fully explained how job replacement anxiety affects job crafting. Drawing on conservation of resources theory, the study develops a moderated dual-path mediation model to examine the relationships between job replacement anxiety and approach and avoidance job crafting from a motivational perspective. Promotion focus and prevention focus are introduced to explain the underlying psychological transformation processes. The study further investigates the moderating role of AI literacy. The empirical analysis was based on three-wave matched survey data from 583 Chinese employees with a broadly balanced gender distribution. The results indicate that job replacement anxiety was indirectly and negatively associated with approach job crafting through lower promotion focus, while an opposing positive direct association resulted in a nonsignificant overall association. For avoidance job crafting, job replacement anxiety had a positive association through higher prevention focus. AI literacy buffered the effects of job replacement anxiety on both forms of regulatory focus and weakened the corresponding indirect effects. The findings suggest that job replacement anxiety is linked to employee job crafting through differentiated motivational pathways and identify AI literacy as an important boundary condition for explaining employees’ adaptation to AI-driven change. These findings advance job crafting research and AI anxiety research by focusing on the job replacement anxiety dimension and offer implications for employee adaptation during AI-driven organizational transformation.

## 1. Introduction

Artificial intelligence (AI), a key driver of technological revolution and industrial transformation, is reshaping job content, skill requirements, and the occupational landscape (Jiang et al., 2024; Meng, 2024). A global survey found that the organizational use of AI has expanded rapidly worldwide (McKinsey, 2024). As AI becomes increasingly embedded in organizations, its impact on the labor market exhibits complex and dual effects, involving both job creation and job displacement, with substitution emerging as the predominant overall impact (Qin et al., 2026). Employees therefore must adapt not only to changing job tasks and skill requirements (Pan et al., 2024), but also to growing uncertainty surrounding their professional value, job stability, and future career prospects. Such uncertainty has contributed to increasing concern about AI-induced job replacement (J. Li & Huang, 2020; Y.-Y. Wang & Wang, 2022). Understanding how employees cope with job replacement anxiety is crucial for explaining employee adaptation behaviors and organizational development in the era of AI.

Job replacement anxiety, as a subdimension of AI anxiety, essentially stems from individuals’ anticipation of potential loss of key resources. It directly refers to concerns that job stability, occupational identity, and income security may be undermined or even that jobs may be replaced by AI (Brougham & Haar, 2018; Fu & Xu, 2025). Conservation of resources theory posits that when critical resources are threatened with potential loss, individuals experience stress responses and adopt either resource-protection or resource-acquisition strategies (Hobfoll, 1989). Accordingly, when employees worry that their jobs may be replaced by AI, what they perceive is not merely technological change itself, but the risk of losing career resources as a result of AI. This raises a critical question: when faced with job replacement anxiety, how will employees reconfigure their work? Will such anxiety weaken their willingness to actively explore and acquire resources, or will it motivate them to adopt new work adaptation strategies?

Job crafting provides an important entry point for understanding the aforementioned issues and serves as a key behavioral strategy through which employees adapt to AI-embedded organizational contexts (He et al., 2024; Huo et al., 2025). Job crafting refers to employees’ self-initiated efforts to alter the task, cognitive, and relational boundaries of their work in order to better align their abilities, preferences, and needs with the work environment. It can be divided into approach job crafting and avoidance job crafting (Bruning & Campion, 2018; Tims et al., 2013; Wrzesniewski & Dutton, 2001). These two forms of behavior reflect the distinct logics of resource acquisition and resource protection, respectively, and can be viewed as differentiated adaptive strategies adopted by employees under uncertainty. In the context of AI replacement pressure, job crafting may involve both proactive expansion and defensive avoidance as distinct coping responses (Huo et al., 2025; Seppälä et al., 2020; F. Zhang et al., 2025). Treating job crafting as an outcome variable therefore helps reveal how employees reconfigure work-related resources to adapt or protect themselves when confronted with job replacement anxiety.

Although existing research has begun to examine how AI-related work conditions shape employees’ job crafting and psychological responses (Cai & Yu, 2024; Gui et al., 2024; He et al., 2024; Mo et al., 2024; Wu et al., 2025a; X. Zhang & Zhao, 2026), recent studies have further focused on specific technological characteristics and psychological mechanisms. For example, AI transparency has been shown to influence employees’ job crafting (Shi et al., 2026), while AI-related system characteristics can have differential effects on job anxiety through AI stress (Fang et al., 2026). A parallel stream of research has examined individuals’ responses to AI from a technology-acceptance perspective. The Technology Acceptance Model (TAM) emphasizes perceived usefulness and perceived ease of use as determinants of technology attitudes and behavioral intentions (Davis, 1989). However, recent findings suggest that favorable evaluations of AI and adverse psychological responses can coexist. Perceived usefulness of generative AI may increase dependence on AI and thereby contribute to fear of AI, while AI literacy can mitigate this process (Liu, 2026). Cross-cultural evidence among higher-education students further indicates that the relationships among perceived usefulness, ease of use, attitudes, and behavioral intentions toward AI can vary across cultural groups (D. Ma et al., 2024). Together, these findings suggest that understanding adaptation to AI requires considering not only whether individuals accept or use AI, but also how they respond to its potential consequences for valued personal and occupational resources.

Against this broader literature, research on AI anxiety and job crafting can be further advanced in two respects. First, although recent literature has linked AI anxiety to job crafting (L. Ma & Li, 2025; Y. Zhang & Chen, 2024), job crafting has often been examined as an intermediary in longer outcome chains, focusing primarily on how AI contexts influence innovation, performance, or career adaptation. Comparatively less attention has been paid to how AI anxiety is translated into different forms of job crafting. Second, AI anxiety is frequently examined as a holistic construct encompassing multiple dimensions, such as learning, job replacement, sociotechnical blindness, and ethical and privacy concerns. This approach has paid insufficient attention to job replacement anxiety, the dimension most directly related to job security. Focusing specifically on this dimension therefore allows a more targeted examination of employees’ psychological and behavioral responses to perceived AI replacement threats.

Conservation of resources theory suggests that individuals’ resource endowments significantly shape their stress appraisal and coping strategies. Compared with general personal resources, AI literacy is a capability-based resource directly oriented toward AI-driven technological change. As an important resource, it refers to individuals’ ability to understand, use, and evaluate AI technologies, encompassing dimensions such as cognitive understanding, application, evaluation, and ethics (B. Wang et al., 2023). AI literacy is highly aligned with the source of job replacement anxiety and exhibits stronger contextual relevance. Employees with higher AI literacy can better understand the potential applications and limitations of AI and more accurately assess its actual impact on job tasks and career development. AI literacy can also enhance employees’ sense of control and self-efficacy when facing AI-driven change (Carolus et al., 2023; Ng et al., 2024). Therefore, employees with higher AI literacy may be more likely to reappraise job replacement anxiety as a manageable career challenge rather than becoming dominated by expectations of resource loss and defensive coping.

While technology-acceptance research helps explain how individuals evaluate and adopt AI, the present study addresses a different but complementary question: how employees adapt when AI is perceived as a threat to valued career resources. Accordingly, conservation of resources theory serves as its primary explanatory framework. This study conceptualizes job replacement anxiety as an anticipatory response to potential resource loss, and regards job crafting as an important behavioral strategy of reconfiguring work-related resources to cope with career threats. It develops a moderated dual-path mediation model to examine how job replacement anxiety influences approach job crafting and avoidance job crafting. By focusing on changes in motivational orientations, the study further explains why such perceived resource threats may lead to divergent resource-related coping behaviors, while also examining the boundary role of AI literacy. This study helps clarify how job replacement anxiety is translated into different work adjustment behaviors, extends job crafting research in the AI context, and also provides a more nuanced understanding of how employees adapt to AI-driven organizational transformation.

## 2. Theoretical Foundations and Research Hypotheses

### 2.1. Theoretical Framework

Conservation of resources theory provides an integrative framework for explaining the resource-threatening nature of job replacement anxiety, the resource-based coping function of job crafting, and the personal resource role of AI literacy. Specifically, job replacement anxiety reflects employees’ anticipation that key career-related resources, such as job stability, occupational identity, skill value, and future development opportunities, may be compromised. Job crafting, in turn, represents a behavioral strategy through which employees reconfigure resources by adjusting their work boundaries in response to resource threats. AI literacy, as an important capability-based resource that enables employees to understand, use, and evaluate AI technologies, may shape their appraisal of AI replacement pressure and subsequent coping responses. Accordingly, conservation of resources theory provides the overarching logic for explaining how job replacement anxiety may lead to different forms of job crafting and how AI literacy may shape this process.

Conservation of resources theory further provides a foundational framework for explaining the relationship between job replacement anxiety and job crafting. The theory emphasizes that individuals strive to acquire, conserve, and protect valued resources and experience stress when these resources are threatened with potential or actual loss (Hobfoll, 1989; Hobfoll et al., 2018). In organizational contexts, such resources include not only resources such as time, energy, and income, but also resources closely related to employees’ sustained adaptation, such as job security, skill value, identity, development opportunities, and psychological energy (Hobfoll et al., 2018). As AI becomes increasingly embedded in organizational processes, employees’ concerns about job stability, occupational identity, skill value, and future development opportunities may therefore reflect anticipated threats to key career-related resources (Bruning & Campion, 2018; Y.-Y. Wang & Wang, 2022). From this perspective, job replacement anxiety represents a response to anticipated resource loss, whereas job crafting serves as a behavioral strategy through which employees readjust their work and reconfigure resources when facing resource threats (Bruning & Campion, 2018; Hobfoll et al., 2018).

Conservation of resources theory also provides a basis for explaining the boundary role of AI literacy. Individuals’ resource endowments shape how they appraise stressful situations and the coping strategies available to them (Hobfoll et al., 2018). AI literacy represents a context-specific personal resource that enables employees to understand, use, and evaluate AI technologies (Carolus et al., 2023; B. Wang et al., 2023). AI literacy can help employees more accurately assess the capabilities and limitations of AI, the scope of its impact on their job roles, and the potential for human–AI complementarity (He et al., 2024; Ng et al., 2024). It may reduce excessive perceptions of resource loss caused by technological uncertainty (Liu, 2026). Consequently, AI literacy may shape how strongly job replacement anxiety is translated into employees’ subsequent motivational and behavioral responses.

### 2.2. Job Replacement Anxiety and Job Crafting

As AI becomes increasingly embedded in organizations, employees are confronted not only with changes in work technologies and tools but also with the continuous restructuring of job tasks, competency requirements, and role value. Existing research has shown that AI contexts significantly influence employees’ job adjustment behaviors (Liang et al., 2026). For example, organizational AI readiness can affect employees’ job crafting through different pathways (X. Zhang & Zhao, 2026); employees’ challenge and hindrance appraisals of AI may further influence their job crafting, job insecurity, and service performance (He et al., 2024); and the intelligence of AI assistants may also lead employees to develop different forms of job crafting (Cai & Yu, 2024). In addition, research on AI awareness suggests that employees’ awareness of AI’s impact may motivate them to cope with changes in the technological environment through job crafting (Mo et al., 2024). These studies indicate that, in AI-driven workplace transformation, job crafting has become an important behavioral strategy through which employees respond to technological change, adjust work boundaries, and maintain work adaptation.

Less is known about how employees engage in job crafting under the more threatening condition in which their jobs may be replaced by AI. Existing research on AI anxiety suggests that AI anxiety does not necessarily lead only to negative outcomes. It may have a double-edged effect on employees’ innovative behavior (Y. Zhang & Chen, 2024), and may also influence the innovative behavior of young employees through mechanisms such as job crafting (L. Ma & Li, 2025; Wu et al., 2025b). This implies that, when facing AI-induced anxiety, employees may not simply endure pressure passively, but may also seek adaptation by reconfiguring their work.

From this perspective, the effect of job replacement anxiety on job crafting is likely to be complex. Job crafting under AI replacement pressure is not a unidirectional behavioral response; rather, it may simultaneously reflect expectations of resource loss and needs for resource protection and acquisition. Specifically, when employees worry that their jobs may be replaced by AI, they tend to pay greater attention to potential losses related to job security, skill obsolescence, and restricted future development. Under such expectations of resource loss, employees may become more inclined to conserve existing resources and reduce their proactive investment in new tasks, new skills, and opportunities for human–AI collaboration. As a result, their willingness to enhance work adaptation by expanding task boundaries, acquiring new resources, and taking on challenging demands may be weakened. This is inconsistent with the logic of resource acquisition and proactive expansion emphasized by approach job crafting (Bruning & Campion, 2018).

At the same time, job replacement anxiety may also be associated with greater attention to risk control and resource protection. Employees may seek to reduce further resource depletion by cutting back on highly demanding tasks, avoiding work demands characterized by high uncertainty, or narrowing their role boundaries. This aligns with the logic of resource protection and stress avoidance emphasized by avoidance job crafting (Bruning & Campion, 2018). Accordingly, the following hypotheses are proposed:

**H1.**  *Job replacement anxiety is negatively related to approach job crafting and positively related to avoidance job crafting.*

**H1a.**  *Job replacement anxiety is negatively related to approach job crafting.*

**H1b.**  *Job replacement anxiety is positively related to avoidance job crafting.*

### 2.3. The Mediating Role of Regulatory Focus

Conservation of resources theory explains why employees develop needs for resource protection or resource acquisition when their resources are threatened. However, when employees perceive that their jobs may be replaced by AI, they do not automatically adopt a fixed mode of work adjustment. Their subsequent behavioral orientation also depends on the specific motivational focus activated under threatening conditions. Although regulatory focus may reflect relatively stable individual tendencies, it can also be situationally activated by environmental cues and perceived threats (Y. Chen et al., 2024; Johnson et al., 2017; Koopmann et al., 2016; Lai et al., 2018; L. Li et al., 2010).

Promotion focus and prevention focus represent distinct rather than mutually exclusive motivational orientations. Promotion focus emphasizes growth, ideals, and gain acquisition, and is associated with achieving positive outcomes. Prevention focus emphasizes safety, responsibility, and loss avoidance, and is associated with preventing negative outcomes through caution, conservatism, and risk avoidance (Higgins, 1997, 1998). These two orientations correspond to the coping logics of resource acquisition and resource protection, respectively, and are consistent with the behavioral characteristics of approach job crafting and avoidance job crafting (Bruning & Campion, 2018). Under AI replacement pressure, changes in these motivational orientations may therefore help explain how job replacement anxiety is translated into different forms of job crafting.

Accordingly, the following hypothesis is proposed:

**H2.**  *Regulatory focus mediates the relationship between job replacement anxiety and job crafting through distinct promotion- and prevention-focus pathways.*

On the resource acquisition path, promotion focus explains why job replacement anxiety may weaken employees’ approach to job crafting. Promotion focus reflects individuals’ concern for growth, ideals, and gain acquisition. Employees with high promotion focus are generally more inclined to enhance their capabilities and achieve goals through proactive exploration, opportunity seeking, and resource investment. Existing research has shown that challenge stressors can activate employees’ promotion focus and further enhance their creative performance (J. Wang et al., 2020). Employees with higher promotion focus are also more likely to adopt proactive ways of working to satisfy their needs for growth and development (Y. Wang & Zhu, 2021).

Under AI replacement pressure, employees may focus their attention on whether their jobs remain secure, whether their skills are becoming obsolete, and whether their career prospects are becoming constrained. As a result, their attention to growth opportunities, career achievement, and positive gains may be weakened. Conservation of resources theory suggests that when individuals perceive threats to key resources, they tend to prioritize resource preservation and risk avoidance while reducing investment in resource-building behaviors. Therefore, job replacement anxiety may constrain employees’ positive expectations about future development, making it more difficult for them to maintain promotion focus centered on growth and gain acquisition.

Lower promotion focus may, in turn, be associated with less approach job crafting. Approach job crafting emphasizes employees’ proactive efforts to increase job resources, expand task boundaries, seek challenging job demands, and improve person-job fit by actively changing job content and work relationships (Bruning & Campion, 2018). Although promotion focus and approach job crafting share an approach- and growth-oriented direction, they represent different components of the motivational process: promotion focus reflects an internal orientation toward gains and advancement, whereas approach job crafting refers to enacted changes in employees’ work roles, tasks, technologies, and social interactions. Because promotion focus centers on growth, progress, and the realization of the ideal self, employees with higher promotion focus are more likely to view work changes as development opportunities and to acquire new resources, enhance their capabilities, and expand their role boundaries through job crafting. Prior research has also shown that promotion focus can significantly promote employees’ proactive behavior and facilitate more active adaptation to work demands through job crafting (W. Chen et al., 2023). Thus, when job replacement anxiety weakens employees’ promotion focus, their motivation to achieve self-improvement by expanding task boundaries, proactively seeking resources, and pursuing challenges is also likely to decline, thereby reducing approach job crafting. Accordingly, the following hypothesis is proposed:

**H2a.**  *Job replacement anxiety is indirectly and negatively associated with approach job crafting through lower promotion focus.*

On the resource protection path, prevention focus explains why job replacement anxiety may strengthen employees’ avoidance job crafting. Prevention focus reflects individuals’ concern for safety, responsibility, and loss avoidance. Employees with higher prevention focus are more attentive to potential risks and negative outcomes and are more inclined to avoid losses through cautious, conservative, and avoidance-oriented behaviors. Unlike promotion focus, which emphasizes gain acquisition, prevention focus is more likely to be activated in threatening situations. When individuals perceive that the external environment may undermine their safety, responsibility fulfillment, or stable state, their attention shifts more strongly toward loss prevention and risk control (Crowe & Higgins, 1997).

Importantly, job replacement anxiety and prevention focus capture different psychological processes despite their close theoretical relationship. Job replacement anxiety reflects an anxiety response to a specific employment-related threat posed by AI, whereas prevention focus reflects a self-regulatory motivational orientation that directs attention toward security, vigilance, and loss avoidance. Job replacement anxiety may activate employees’ prevention focus by heightening concerns about skill devaluation, occupational identity, and future income. The risk of AI replacement may not only weaken employees’ confidence in job stability, but also increase their concerns about skill devaluation, damage to occupational identity, and uncertainty regarding future income. In this context, employees are more likely to interpret AI-driven change as a threat to existing career-related resources rather than merely as a development opportunity, thereby intensifying their focus on risk, loss, and responsibility fulfillment. Existing research has shown that hindrance stressors can induce prevention focus, leading employees to pay greater attention to risk avoidance and loss prevention (J. Wang et al., 2020). Complex work situations, such as multitasking, may also enhance prevention focus and lead employees to display more negative or avoidant responses (Jiao et al., 2024). Accordingly, job replacement anxiety may strengthen employees’ concern for safety and loss, thereby activating prevention focus.

Higher prevention focus may, in turn, be associated with greater avoidance job crafting. Avoidance job crafting emphasizes protecting personal resources by reducing hindering job demands, lowering resource consumption, avoiding stressors, or narrowing role boundaries (Bruning & Campion, 2018). When employees are in a state of high prevention focus, their behavioral goals are not to actively acquire additional resources or pursue greater growth, but rather to avoid mistakes, reduce risks, and maintain a sense of security. Therefore, employees with high prevention focus are more likely to cope with stress by reducing effort, avoiding high-risk tasks, or lowering work demands. Prior research has found that employees with a high prevention focus tend to satisfy their safety needs through conservative and avoidance-oriented strategies (F. Zhang et al., 2025). Under stressful conditions, prevention focus may also drive employees to adopt defensively oriented job crafting behaviors (Hou et al., 2022). Thus, job replacement anxiety may guide employees toward avoidance job crafting oriented toward resource conservation and risk avoidance by enhancing prevention focus. Accordingly, the following hypothesis is proposed:

**H2b.**  *Job replacement anxiety is indirectly and positively associated with avoidance job crafting through higher prevention focus.*

### 2.4. The Moderating Role of AI Literacy

Building on the resource-based perspective outlined above, AI literacy represents a capability-based personal resource comprising the knowledge, skills, and cognitive abilities needed to understand, evaluate, and engage with AI technologies (Hobfoll, 1989; B. Wang et al., 2023). Existing research has shown that AI-related knowledge can influence employees’ challenge-hindrance appraisals of AI and their subsequent work responses (He et al., 2024), and that AI literacy significantly affects individuals’ willingness to adopt generative AI (Yu et al., 2026). Higher AI literacy enables employees to more accurately understand the operating logic, application boundaries, and potential risks of AI technologies, and to use AI tools more effectively to solve problems in actual work settings (H. Li & Zhao, 2025). These cognitive and skill-based resources allow employees to assess job displacement risks more rationally, recognize that AI does not simply replace jobs but often involves task restructuring, skill upgrading, and opportunities for human–AI complementarity, and believe that they can adapt to new roles through learning and adjustment (Tan & Wang, 2025). Taken together, these capabilities may shape how employees appraise AI replacement pressure and, consequently, how job replacement anxiety is translated into regulatory focus and subsequent job crafting. Accordingly, the following hypothesis is proposed:

**H3.**  *AI literacy moderates the process through which job replacement anxiety is associated with job crafting via regulatory focus.*

AI literacy may buffer the inhibitory effect of job replacement anxiety on promotion focus. Job replacement anxiety reflects employees’ concerns that AI may undermine their job stability, occupational identity, skill value, and future development opportunities. This anticipation of resource loss may lead employees to focus more on whether their jobs remain secure, whether their skills are becoming obsolete, and whether their future career prospects are becoming constrained, thereby weakening their attention to growth opportunities, positive outcomes, and resource gains. When employees have low AI literacy, their lack of knowledge and skills regarding AI technologies and their work-related application boundaries makes them more likely to perceive AI as an uncontrollable replacement threat. This may amplify the perceived resource loss triggered by job replacement anxiety, making it more difficult for them to maintain promotion focus centered on growth, gain acquisition, and the attainment of positive goals. In contrast, higher AI literacy provides employees with critical capability-based resources for understanding, evaluating, and using AI. Employees with higher AI literacy are therefore more likely to assess job displacement risks rationally and recognize that learning AI-related knowledge, adjusting work methods, and exploring human–AI collaboration may still enable resource replenishment and capability enhancement. Conservation of resources theory suggests that when individuals possess sufficient reserves of key resources, they are less likely to shift their attention entirely toward resource defense when facing threatening situations; instead, they are more likely to retain the possibility of resource investment and resource acquisition (Hobfoll, 1989). Therefore, when AI literacy is high, employees may maintain their focus on growth opportunities and positive outcomes even when experiencing job replacement anxiety, thereby weakening the negative effect of job replacement anxiety on promotion focus. Accordingly, the following hypothesis is proposed:

**H3a.**  *AI literacy moderates the relationship between job replacement anxiety and promotion focus, such that the negative association of job replacement anxiety on promotion focus is weaker when AI literacy is higher.*

Given that promotion focus serves as a key mediating mechanism through which job replacement anxiety influences approach job crafting, the buffering effect of AI literacy on the relationship between job replacement anxiety and promotion focus may further shape the corresponding indirect effect. Because AI literacy can weaken the negative effect of job replacement anxiety on promotion focus, the indirect effect of job replacement anxiety on approach job crafting through promotion focus should also become weaker as AI literacy increases. Accordingly, the following hypothesis is proposed:

**H3b.**  *AI literacy moderates the indirect association of job replacement anxiety on approach job crafting through promotion focus, such that the negative indirect effect becomes weaker when AI literacy is higher.*

AI literacy may also weaken the activating effect of job replacement anxiety on prevention focus. Arising from a perceived threat to the core resource of job security (Brougham & Haar, 2018; Fu & Xu, 2025), job replacement anxiety heightens individuals’ sensitivity to resource loss and increases their tendency toward risk avoidance, thereby activating prevention focus oriented toward responsibility fulfillment, error avoidance, and loss minimization. As a transferable capability-based resource, AI literacy, on the one hand, enables employees to develop a deeper understanding of AI and more accurately assess the actual likelihood and scope of job displacement risks, thereby reducing excessive threat perceptions related to AI replacement. On the other hand, AI literacy provides employees with more concrete coping resources, enhancing their sense of control and self-efficacy in addressing AI-related threats (Carolus et al., 2023; Ng et al., 2024). Under the logic of conservation of resources theory, individuals with more abundant resources tend to experience weaker stress responses when facing threats, because they are more capable of regaining resources through active adjustment rather than relying primarily on defensive strategies to maintain the status quo. Therefore, when AI literacy is high, the positive effect of job replacement anxiety on prevention focus may be attenuated. Accordingly, the following hypothesis is proposed:

**H3c.**  *AI literacy moderates the relationship between job replacement anxiety and prevention focus, such that the positive association of job replacement anxiety on prevention focus is weaker when AI literacy is higher.*

Given that prevention focus serves as a key mediating mechanism through which job replacement anxiety influences avoidance job crafting, the weakening effect of AI literacy on the relationship between job replacement anxiety and prevention focus may further shape the corresponding indirect effect. Because AI literacy may help reduce the positive effect of job replacement anxiety on prevention focus, the indirect effect of job replacement anxiety on avoidance job crafting through prevention focus should also become weaker as AI literacy increases. Accordingly, the following hypothesis is proposed:

**H3d.**  *AI literacy moderates the indirect effect of job replacement anxiety on avoidance job crafting through prevention focus, such that the positive indirect association becomes weaker when AI literacy is higher.*

**Figure 1:**
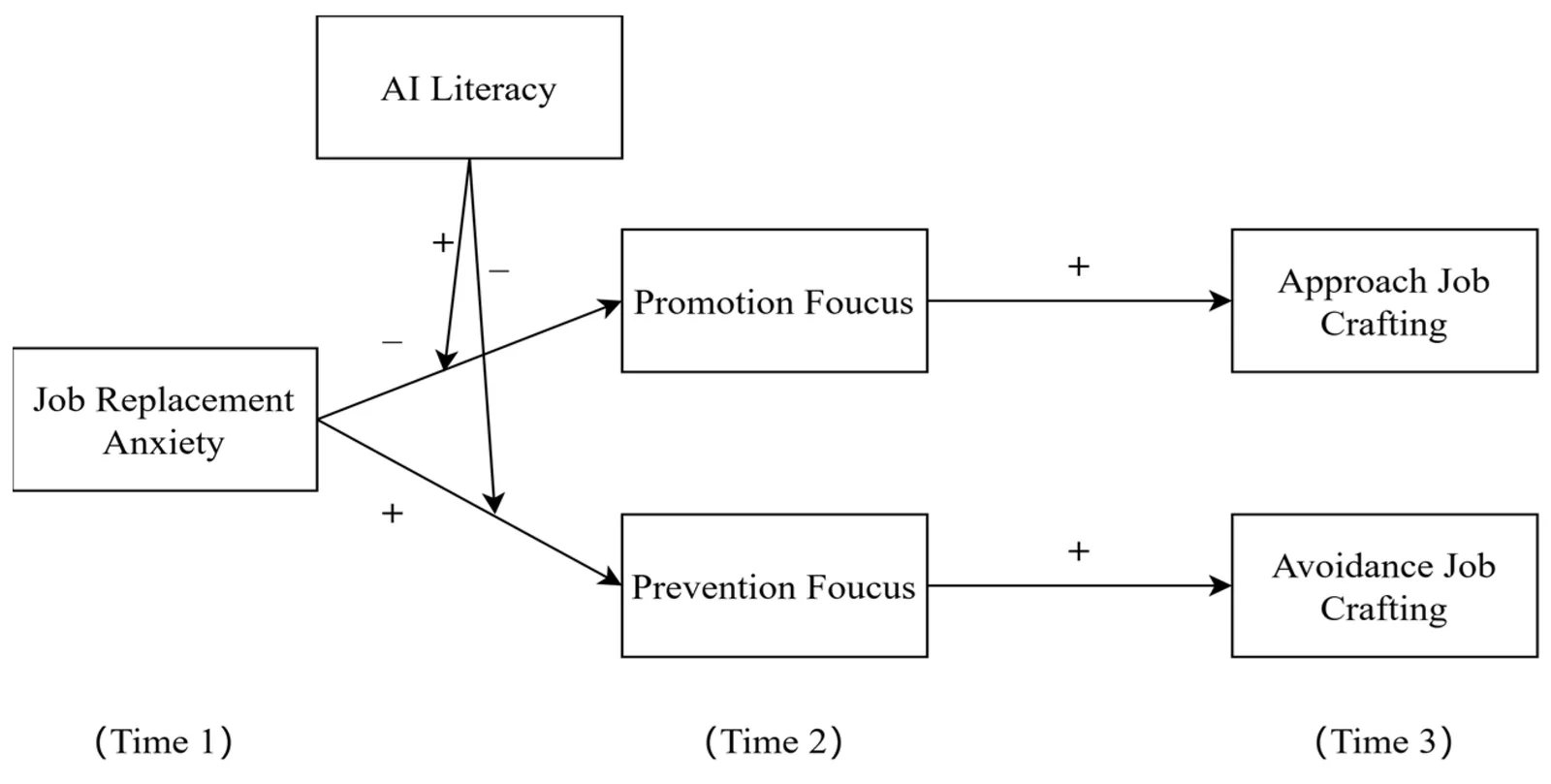
Research model.

In summary, grounded in conservation of resources theory, this study examines the mechanisms through which job replacement anxiety influences approach job crafting and avoidance job crafting via promotion focus and prevention focus, respectively, and clarifies the boundary role of AI literacy. The research model is shown in Figure 1.

## 3. Methods

### 3.1. Sample and Procedures

As the survey was administered in Chinese, a translation and back-translation procedure was conducted to ensure linguistic and cultural appropriateness. Two researchers were involved in the translation process. The original items were translated into Chinese and subsequently back-translated into English. Discrepancies between the original and back-translated versions were discussed and resolved with the assistance of a professor to preserve the intended meaning of the items. Before the formal investigation, a pilot survey was conducted with 70 participants to assess the clarity of the questionnaire items, the applicability of the selected measurement scales, and the response burden of the questionnaire. Participants in the pilot survey were not included in the formal sample. The pilot results indicated that the questionnaire items were generally clear and understandable and that the selected scales were applicable to the study context. Based on participants’ feedback, minor wording adjustments were made to improve the clarity and readability of the questionnaire without changing the substantive content of the measurement items.

During the formal survey stage, this study distributed questionnaires through both online and offline channels to diversify the sample sources. All questionnaires were completed electronically through Questionnaire Star to reduce missing responses and invalid answers. For the online survey, questionnaire links were mainly distributed through WeChat, inviting respondents with work experience to participate. The survey also encouraged them to forward the links to other employees in their professional networks, expanding sample coverage through the snowball sampling strategy. For the offline survey, the research team contacted persons in charge of human resources in multiple organizations. After the research purpose and questionnaire instructions were fully explained, these persons assisted in circulating the electronic survey link to employees and organizing employees to participate in the survey. Before completing the questionnaire through both online and offline surveys, participants were informed of the research purpose, the use of their data, the voluntary nature of their participation, and their right to withdraw. Submission of the questionnaire was regarded as informed consent, and the survey platform restricted repeated submissions from the same IP address.

To reduce common method bias, this study adopted a three-wave matched design for data collection. In the first wave (Time 1), job replacement anxiety, AI literacy, and basic demographic information were measured. In the second wave (Time 2), conducted two weeks later, promotion focus and prevention focus were measured. In the third wave (Time 3), conducted another two weeks later, approach job crafting and avoidance job crafting were measured. The three waves of questionnaires were primarily matched using the last four digits of respondents’ mobile phone numbers. Complete mobile phone numbers were not collected. When duplicate codes occurred, the user IDs recorded by Questionnaire Star were checked to resolve the conflicts. To protect participants’ privacy, after matching was completed, only the successfully matched and de-identified dataset was retained for analysis. The retained dataset was stored on a password-protected device and was accessible only to the research team.

A total of 739 questionnaires were received in the first wave, and 697 valid responses were obtained after invalid questionnaires were excluded. In the second wave, follow-up data collection was conducted based on the valid sample from the first wave; 679 questionnaires were returned, yielding 652 valid responses. In the third wave, follow-up data collection continued, and 603 questionnaires were returned. Ultimately, 583 valid matched questionnaires were obtained, with a valid matching rate of 78.89%. According to DeVellis and Thorpe (DeVellis & Thorpe, 2022), approximately 5–10 respondents per item is a commonly used practical guideline. The study included 62 scale items in total, and the final matched sample of 583 fell within this recommended range. Based on the final analytic sample using G*Power 3.1 (Faul et al., 2009), a sensitivity power analysis was further conducted for the focal regression effect. With *N* = 583, α = 0.05, statistical power = 0.80, one tested predictor, and seven total predictors, the minimum detectable incremental effect size was f^2^ = 0.014. Thus, the final sample had 80% power to detect incremental effects of this magnitude or larger. To ensure data quality, this study adopted multiple quality control procedures. First, attention-check items were included to identify careless responses. Second, questionnaires completed in less than 90 s were excluded. Finally, questionnaires showing obvious regular response patterns were manually screened, and suspicious responses, such as consecutive selection of the same option, were removed. Only questionnaires that passed the quality screening and were successfully matched across all three waves were retained for subsequent statistical analysis.

The final sample consisted of employees from various industries in China. The descriptive statistics of the sample are presented in Table 1. Overall, the sample exhibits a certain degree of heterogeneity in terms of gender, age, education level, and job position. In addition, most respondents had some experience using AI, with a relatively high proportion reporting frequent or very frequent use. This is well aligned with the research context and provides an appropriate data basis for subsequent hypothesis testing.

### 3.2. Measurement

This study examined six core variables: job replacement anxiety, regulatory focus (including promotion focus and prevention focus), job crafting (including approach job crafting and avoidance job crafting), and AI literacy. All variables were measured using well-established scales that have been widely validated in prior research. Job replacement anxiety, regulatory focus, and AI literacy were rated on five-point agreement scales ranging from 1 (strongly disagree) to 5 (strongly agree), whereas job crafting items were rated on five-point frequency scales ranging from 1 (almost never) to 5 (always). Representative items for each focal construct and each of its (sub-)dimensions or content domains are reported in Table A1 in Appendix A.

(1) Job replacement anxiety: Job replacement anxiety was measured using the relevant subscale of the AI anxiety scale developed by Wang and Wang (Y.-Y. Wang & Wang, 2022). The scale consists of six items. The Cronbach’s α coefficient for this scale was 0.917.
(2) Regulatory focus: promotion focus and prevention focus were measured using the Work Regulatory Focus Scale developed by Neubert et al. (Neubert et al., 2008). They are two nine-item subscales of the same instrument. The promotion focus scale reflects gains, achievement, and ideals, whereas the prevention focus reflects security, oughts, and losses. The Cronbach’s α coefficient was 0.901 for promotion focus and 0.921 for prevention focus.
(3) Job crafting: Approach and avoidance job crafting were measured using the items from the scale developed by Bruning and Campion (Bruning & Campion, 2018). The approach job crafting scale includes four dimensions, namely work role expansion, technology adoption, social expansion, and metacognition, with a total of 19 items. Avoidance job crafting was assessed with seven items covering withdrawal and work role reduction. The Cronbach’s α coefficient was 0.972 for approach job crafting and 0.908 for avoidance job crafting.
(4) AI literacy: AI literacy was measured using the scale developed by B. Wang et al. (2023). The scale includes four dimensions, namely awareness, use, evaluation, and ethics, with a total of 12 items. The Cronbach’s α coefficient for this scale was 0.899.
(5) Control variables: The selection of control variables should be based on theoretical relevance and prior empirical evidence, so as to exclude potential confounding factors that may influence the relationships among the core variables (Cao et al., 2020). In AI contexts, individual demographic characteristics may affect AI anxiety, attitudes toward technology, and work-related behavioral responses (Hopcan et al., 2024; Kaya et al., 2024; Russo et al., 2025). Job crafting behavior may also be influenced by individual characteristics such as gender (Ji & Chen, 2024; Lim et al., 2025). In addition, the frequency of AI product/technology use reflects employees’ degree of exposure to and experience with AI, which may influence their perceptions of and responses to AI technologies. Although AI use frequency is conceptually related to the usage dimension of AI literacy, the two are not equivalent. AI use frequency captures how often employees use AI products or technologies, whereas the usage dimension of AI literacy reflects their competence in applying AI effectively to accomplish tasks (B. Wang et al., 2023). Thus, controlling for AI use frequency helps distinguish the role of AI literacy from differences attributable simply to the extent of employees’ exposure to and use of AI technologies. Therefore, this study included gender, age, educational attainment, years of work experience, job level, and frequency of AI product/technology use as control variables in the research model.

### 3.3. Statistical Analysis

Statistical analyses were conducted using SPSS 27.0 and AMOS 29.0. The analytical procedure consisted of three stages. First, the reliability and validity of the measurement model and potential common method bias were assessed. Second, descriptive statistics and Pearson correlations were examined to provide preliminary information on the relationships among the focal variables. Third, the hypothesized moderated dual-path mediation model was tested using an integrated latent-variable structural equation model.

Reliability was assessed using Cronbach’s α and composite reliability (CR), with values of approximately 0.70 or higher treated as indicating acceptable internal consistency. Convergent validity was evaluated using average variance extracted (AVE), with values of 0.50 or higher used as the conventional criterion. For job replacement anxiety, which was specified as a first-order construct, CR and AVE were calculated based on its standardized item loadings. For the multidimensional constructs modeled as second-order factors, higher-order CR and AVE were calculated based on the standardized loadings of their respective first-order dimensions on the corresponding second-order constructs. Discriminant validity was assessed using the Fornell–Larcker criterion and the heterotrait–monotrait ratio of correlations (HTMT), with an HTMT value below 0.85 adopted as a conservative criterion (Fornell & Larcker, 1981; Henseler et al., 2015).

Confirmatory factor analysis (CFA) was used to evaluate the measurement structure. A hierarchical six-factor measurement model was specified in which job replacement anxiety was modeled as a first-order latent construct with its six items as indicators, whereas AI literacy, promotion focus, prevention focus, approach job crafting, and avoidance job crafting were modeled as second-order latent constructs based on their respective theoretical dimensions or content domains. Model fit was evaluated jointly using χ^2^/df, the root mean square error of approximation (RMSEA), Tucker–Lewis index (TLI), comparative fit index (CFI), and incremental fit index (IFI). Values around or above 0.90 for TLI, CFI, and IFI and below 0.08 for RMSEA were treated as general indicators of acceptable fit, whereas values approaching 0.95 for the incremental fit indices and approximately 0.06 for RMSEA represent more stringent guidelines (Bentler, 1990; Bentler & Bonett, 1980; Hu & Bentler, 1999). These indices were considered jointly rather than interpreted as rigid universal cutoff rules (Marsh et al., 2004a). χ^2^/df was reported as an additional descriptive indicator of model fit. When alternative measurement models were compared, the Akaike information criterion (AIC) was also considered, with lower values indicating better relative fit among competing models (Akaike, 1974).

Common method bias was assessed using Harman’s single-factor test followed by an unmeasured latent method factor analysis (Podsakoff et al., 2003; Williams et al., 2010). For the latter analysis, the original six-factor measurement structure was retained, and an additional common method factor was introduced. All 62 measurement items were specified to load simultaneously on their corresponding substantive factors and on the common method factor, while the common method factor was specified as uncorrelated with the substantive factors. The fit of this model was compared with that of the original six-factor measurement model. Multicollinearity among the observed predictors and covariates was additionally examined using variance inflation factors (VIFs), with a value of 5 used as a screening benchmark rather than as a rigid cutoff (O’brien, 2007).

Hypothesis testing was conducted using an integrated latent-variable SEM. The two mediating pathways and the moderating effects of AI literacy were estimated simultaneously. Gender, age, educational attainment, years of work experience, job level, and frequency of AI product/technology use were included as control variables. Gender was binary-coded, whereas the remaining control variables were entered using ascending ordinal codes corresponding to their ordered response categories. As a robustness check, the integrated structural model was also re-estimated by replacing the five ordinal control variables with sets of dummy variables. To estimate the latent interaction between job replacement anxiety and AI literacy, each of the two interacting constructs was represented by three parcels, and three matched-pair product indicators were constructed using double-mean centering and specified as indicators of the latent interaction factor. The product-indicator approach and double-mean-centering procedure followed established methods for estimating latent-variable interactions (Lin et al., 2010; Marsh et al., 2004b). Significant interaction effects were further examined using simple slope analyses at one standard deviation below the mean, at the mean, and at one standard deviation above the mean of AI literacy. Indirect effects, conditional indirect effects, and indices of moderated mediation were evaluated using 5000 bootstrap samples with bias-corrected 95% confidence intervals; an effect was regarded as statistically significant when the corresponding confidence interval did not include zero (Preacher & Hayes, 2008).

## 4. Results

### 4.1. Reliability and Validity

The Cronbach’s α coefficients of all variables were above 0.80, indicating good internal consistency. For the multidimensional measures, the dimension or content-domain level Cronbach’s α coefficients ranged from 0.775 to 0.933, as reported in Table A1.

As shown in Table 2, the six-factor model showed an acceptable fit to the data: χ^2^/df = 2.112, RMSEA = 0.044, TLI = 0.923, CFI = 0.927, and IFI = 0.927, all of which reached acceptable thresholds. By contrast, the model fit of the five-factor, four-factor, three-factor, and single-factor models was poorer and had higher AIC values. These results provide support for the discriminant validity of the six core constructs.

The standardized loadings of all measurement items on their respective first-order factors were above 0.600, and the second-order factor loadings of all constructs were above 0.700, further supporting the stability of the measurement structure. The CR values were 0.918 for job replacement anxiety, 0.933 for promotion focus, 0.932 for prevention focus, 0.916 for AI literacy, 0.964 for approach job crafting, and 0.884 for avoidance job crafting, all exceeding 0.80. The corresponding AVE values were 0.652, 0.822, 0.820, 0.732, 0.870, and 0.792, respectively, all exceeding 0.60. These results indicate that all variables had good convergent validity.

Furthermore, consistent with the Fornell–Larcker criterion (Fornell & Larcker, 1981), the square root of the AVE for each core variable was greater than its correlations with the other core variables, providing support for discriminant validity among the variables (Table 3). To provide an additional assessment of discriminant validity, the HTMT was calculated (Henseler et al., 2015). As reported in Table A2, all pairwise HTMT values were below 0.85, ranging from 0.056 to 0.847. The highest HTMT value was observed between promotion focus and approach job crafting (0.847), followed by job replacement anxiety and prevention focus (0.766). Although the former value approached the conservative threshold of 0.85, it remained below this criterion. Taken together with the CFA model comparisons and the conceptual distinctions among the constructs, the HTMT results provide additional support for their empirical distinctiveness, while the relatively strong association between promotion focus and approach job crafting warrants cautious interpretation.

### 4.2. Common Method Bias

Harman’s single-factor test showed that six factors had eigenvalues greater than 1, accounting for 62.872% of the cumulative variance. The first factor explained 33.499% of the variance, which is below the critical threshold of 40% (Podsakoff et al., 2003), suggesting that common method bias was not a serious concern.

The unmeasured latent method factor approach provided further evidence. The model including the common method factor yielded the following fit indices: χ^2^/df = 2.192, RMSEA = 0.045, TLI = 0.918, CFI = 0.924, and IFI = 0.925. Compared with the original six-factor model, the model fit did not improve after the common method factor was added, and some fit indices decreased slightly. These results further indicate that common method bias was unlikely to have seriously affected the conclusions of this study.

### 4.3. Correlation Analysis

Table 3 reports the means, standard deviations, and Pearson correlation coefficients among the six focal constructs based on the observed composite scores of the variables. The results show that job replacement anxiety was not significantly correlated with approach job crafting (r = 0.043, *p* > 0.05), but was significantly positively correlated with avoidance job crafting (r = 0.437, *p* < 0.01). Job replacement anxiety was significantly negatively correlated with promotion focus (r = −0.111, *p* < 0.01) and significantly positively correlated with prevention focus (r = 0.704, *p* < 0.01). Promotion focus was significantly positively correlated with approach job crafting (r = 0.791, *p* < 0.01), while prevention focus was significantly positively correlated with avoidance job crafting (r = 0.527, *p* < 0.01). Multicollinearity diagnostics further showed that all VIF values were below 5, indicating no serious multicollinearity concerns among the observed predictors and covariates. These results provide preliminary evidence regarding the bivariate associations among the focal constructs and provide a descriptive basis for the subsequent structural analyses.

### 4.4. Hypothesis Testing

The integrated structural model showed an acceptable fit to the data: χ^2^(1576) = 3435.602, χ^2^/df = 2.180, RMSEA = 0.045, TLI = 0.925, CFI = 0.931, and IFI = 0.931. The squared multiple correlations were 0.346 for promotion focus, 0.791 for prevention focus, 0.862 for approach job crafting, and 0.464 for avoidance job crafting. All direct, indirect, total, and conditional indirect effects reported below were estimated while accounting for the control variables specified in Section 3.3.

#### 4.4.1. Main Effects and Mediating Effects Analysis

The relationships between job replacement anxiety and the two types of job crafting, together with the mediating roles of promotion focus and prevention focus, were first examined within the integrated structural model. The structural path estimates are presented in Table 4, and the bootstrap estimates of the total and indirect effects are reported in Table 5.

For approach job crafting, the total effect of job replacement anxiety at the mean level of AI literacy was not statistically significant (Effect = −0.072, Boot SE = 0.042, bias-corrected 95% CI = [−0.150, 0.014], *p* = 0.109). The sensitivity analysis reported in Section 3.1 indicated that the final sample had 80% power to detect incremental effects of f^2^ = 0.014 or larger under the specified regression framework; therefore, the possibility of an effect smaller than this benchmark cannot be ruled out. Nevertheless, because H1a predicted an overall negative effect of job replacement anxiety on approach job crafting and the estimated total effect was not statistically significant, H1a was not supported.

The mediation analysis nevertheless revealed a significant negative indirect pathway through promotion focus. Job replacement anxiety was significantly negatively associated with promotion focus (B = −0.187, SE = 0.030, β = −0.267, C.R. = −6.255, *p* < 0.001), whereas promotion focus was significantly positively associated with approach job crafting (B = 1.247, SE = 0.072, β = 0.911, C.R. = 17.290, *p* < 0.001). At the mean level of AI literacy, the resulting indirect effect of job replacement anxiety on approach job crafting through promotion focus was significant (Effect = −0.234, Boot SE = 0.043, bias-corrected 95% CI = [−0.317, −0.149], *p* = 0.001), supporting H2a.

At the same time, after promotion focus and the other variables in the structural model were taken into account, the residual direct effect of job replacement anxiety on approach job crafting was significantly positive (B = 0.162, SE = 0.025, β = 0.168, C.R. = 6.461, *p* < 0.001). Thus, the positive residual direct effect and negative indirect effect operated in opposite directions and substantially offset one another, resulting in a nonsignificant total effect. This pattern is consistent with inconsistent mediation, or a suppression pattern, in which direct and indirect effects have opposite signs (MacKinnon et al., 2000). Therefore, although H1a was not supported at the total-effect level, the results indicate the coexistence of opposing pathways linking job replacement anxiety to approach job crafting.

For avoidance job crafting, the total effect of job replacement anxiety at the mean level of AI literacy was significantly positive (Effect = 0.468, Boot SE = 0.053, bias-corrected 95% CI = [0.361, 0.571], *p* < 0.001), supporting H1b.

Job replacement anxiety had a significant positive effect on prevention focus (B = 0.444, SE = 0.025, β = 0.714, C.R. = 17.501, *p* < 0.001), and prevention focus had a significant positive effect on avoidance job crafting (B = 1.220, SE = 0.135, β = 0.732, C.R. = 9.072, *p* < 0.001). At the mean level of AI literacy, the indirect effect of job replacement anxiety on avoidance job crafting through prevention focus was significant (Effect = 0.542, Boot SE = 0.071, bias-corrected 95% CI = [0.413, 0.694], *p* < 0.001), supporting H2b. In contrast, the residual direct effect of job replacement anxiety on avoidance job crafting was not statistically significant (B = −0.074, SE = 0.074, β = −0.071, C.R. = −0.993, *p* = 0.321).

In summary, H1a was not supported, whereas H1b was supported; therefore, H1 was partially supported. Both H2a and H2b were supported, and H2 was supported.

#### 4.4.2. Moderating Effects Analysis

To examine the moderating role of AI literacy, this study examined the latent interaction between job replacement anxiety and AI literacy. The interaction term had a significant positive effect on promotion focus (B = 0.173, SE = 0.050, β = 0.135, C.R. = 3.431, *p* < 0.001, bias-corrected 95% CI = [0.043, 0.303]), indicating that AI literacy moderated the relationship between job replacement anxiety and promotion focus.

**Figure 2:**
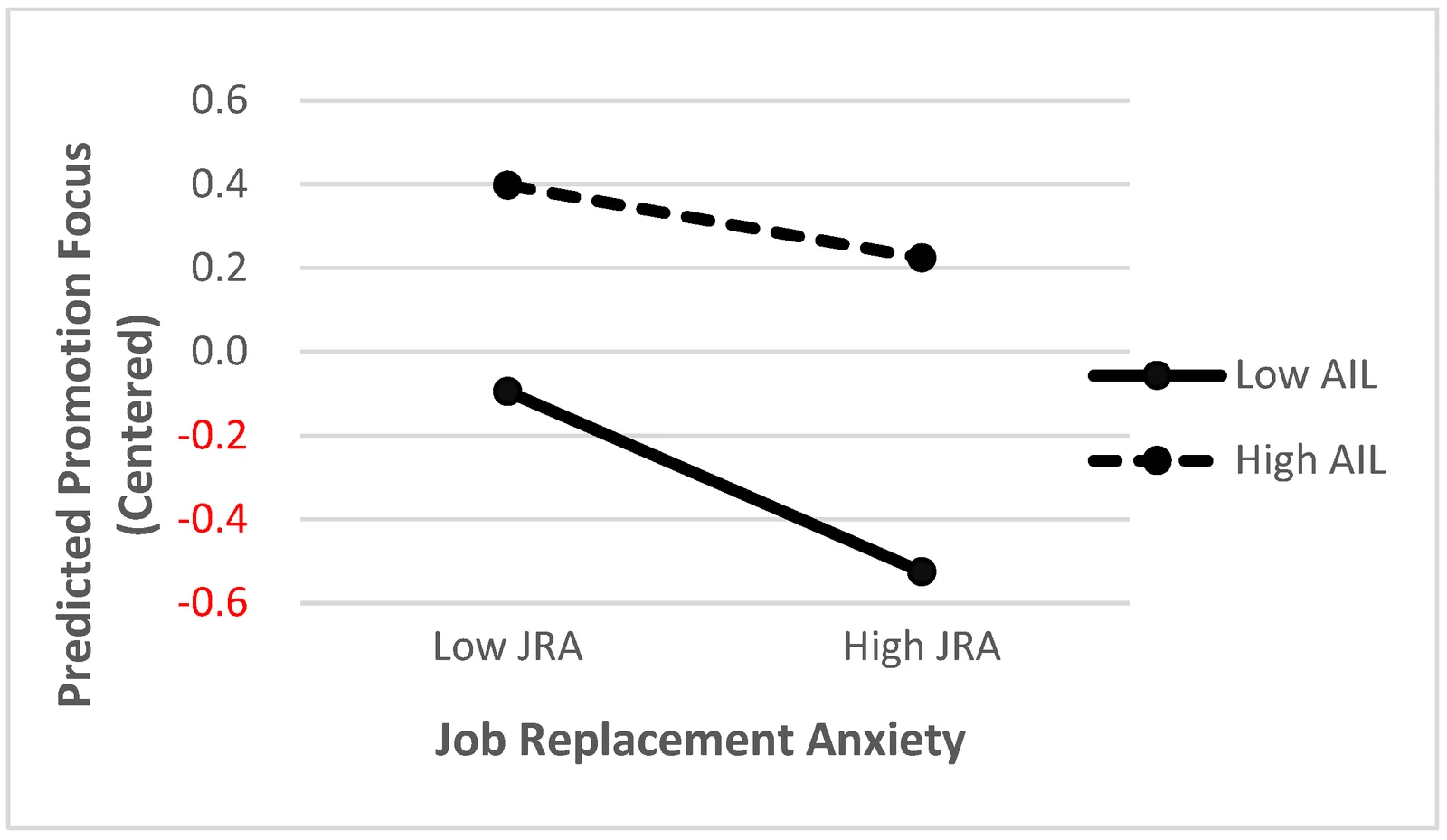
The Moderating Effect of AI Literacy on the Relationship Between Job Replacement Anxiety and Promotion Focus.

To further interpret this interaction, simple slope analyses were conducted at low (M − 1 SD), mean, and high (M + 1 SD) levels of AI literacy (Table 6). When AI literacy was low, job replacement anxiety was significantly negatively associated with promotion focus (Effect = −0.268, Boot SE = 0.054, bias-corrected 95% CI = [−0.373, −0.159], *p* < 0.001). The negative effect remained significant at the mean level of AI literacy (Effect = −0.187, Boot SE = 0.035, bias-corrected 95% CI = [−0.254, −0.120], *p* < 0.001) and at the high level of AI literacy (Effect = −0.107, Boot SE = 0.037, bias-corrected 95% CI = [−0.181, −0.036], *p* = 0.002). However, its magnitude progressively decreased as AI literacy increased. As shown in Figure 2, the negative relationship between job replacement anxiety and promotion focus was therefore stronger at low AI literacy and weaker at high AI literacy. These results support H3a.

**Figure 3:**
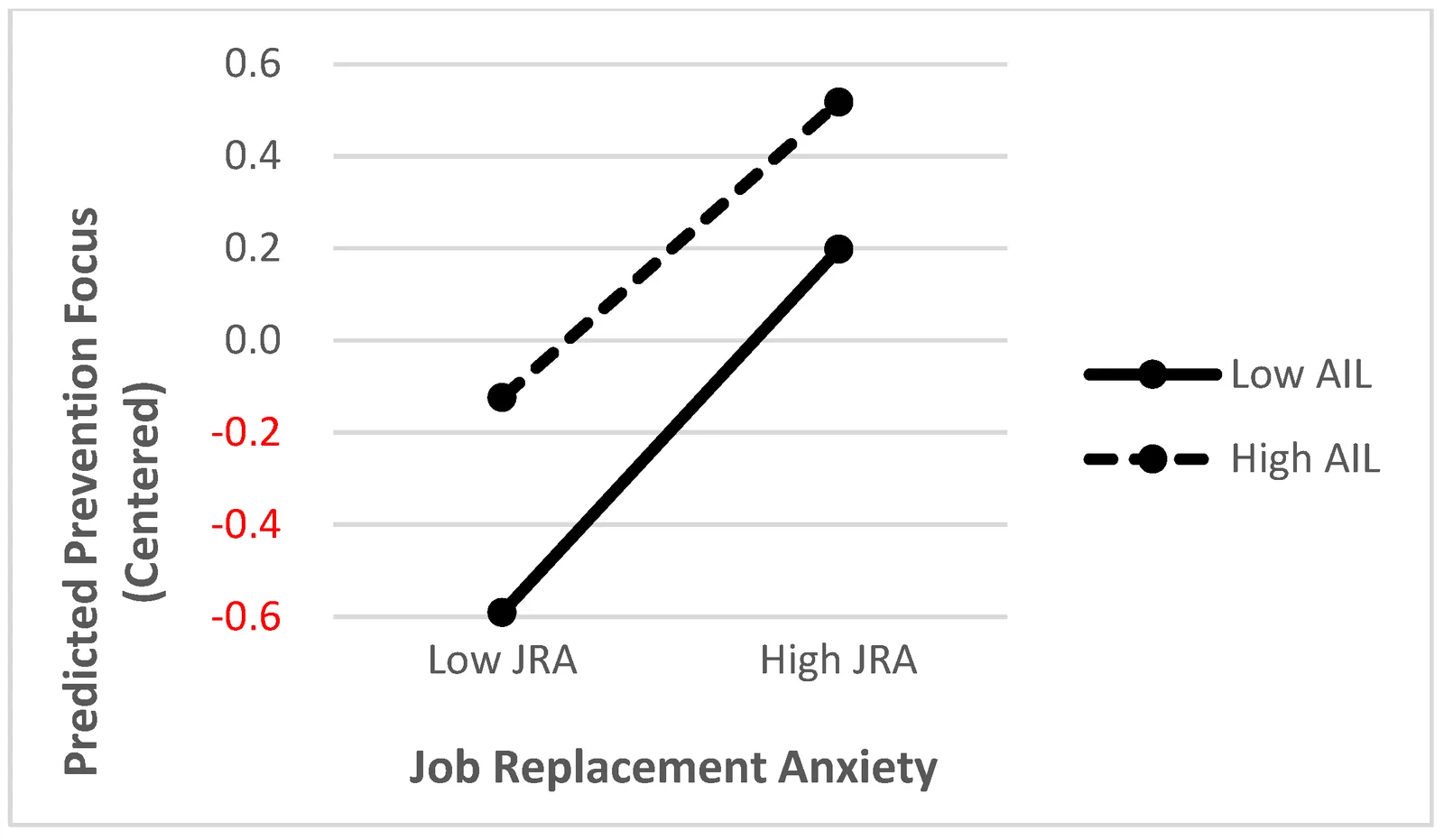
The Moderating Effect of AI Literacy on the Relationship Between Job Replacement Anxiety and Prevention Focus.

The interaction between job replacement anxiety and AI literacy also had a significant negative effect on prevention focus (B = −0.100, SE = 0.031, β = −0.089, C.R. = −3.207, *p* = 0.001, bias-corrected 95% CI = [−0.184, −0.020]). When AI literacy was low, job replacement anxiety had a significant positive effect on prevention focus (Effect = 0.490, Boot SE = 0.039, bias-corrected 95% CI = [0.418, 0.570], *p* < 0.001). The positive effect remained significant at the mean level of AI literacy (Effect = 0.444, Boot SE = 0.026, bias-corrected 95% CI = [0.394, 0.498], *p* < 0.001) and at the high level of AI literacy (Effect = 0.397, Boot SE = 0.025, bias-corrected 95% CI = [0.352, 0.449], *p* < 0.001), but its magnitude decreased as AI literacy increased. As shown in Figure 3, the positive relationship between job replacement anxiety and prevention focus was therefore stronger at low AI literacy and became weaker at high AI literacy. These results support H3c.

#### 4.4.3. Moderated Mediation Effects Analysis

To further examine whether AI literacy moderated the indirect effects of job replacement anxiety on job crafting through regulatory focus, this study estimated the conditional indirect effects at low, mean, and high levels of AI literacy, as reported in Table 7.

For the promotion-focus pathway, the negative indirect effect of job replacement anxiety on approach job crafting was significant at all three levels of AI literacy. When AI literacy was low, the conditional indirect effect was −0.334 (Boot SE = 0.065, bias-corrected 95% CI = [−0.459, −0.202], *p* < 0.001). At the mean level of AI literacy, the conditional indirect effect was −0.234 (Boot SE = 0.043, bias-corrected 95% CI = [−0.317, −0.149], *p* = 0.001). When AI literacy was high, the conditional indirect effect was −0.134 (Boot SE = 0.048, bias-corrected 95% CI = [−0.230, −0.041], *p* = 0.002). The magnitude of the negative indirect effect decreased as AI literacy increased. In addition, the index of moderated mediation was 0.216 (Boot SE = 0.079, bias-corrected 95% CI = [0.056, 0.364], *p* = 0.012). Because the confidence interval did not include zero, H3b was supported.

For the prevention-focus pathway, the positive indirect effect of job replacement anxiety on avoidance job crafting was also significant at all three levels of AI literacy. When AI literacy was low, the conditional indirect effect was 0.599 (Boot SE = 0.080, bias-corrected 95% CI = [0.455, 0.769], *p* < 0.001). At the mean level of AI literacy, the conditional indirect effect was 0.542 (Boot SE = 0.071, bias-corrected 95% CI = [0.413, 0.694], *p* < 0.001). When AI literacy was high, the conditional indirect effect was 0.485 (Boot SE = 0.070, bias-corrected 95% CI = [0.363, 0.641], *p* < 0.001). The magnitude of the positive indirect effect decreased as AI literacy increased. The index of moderated mediation was −0.123 (Boot SE = 0.049, bias-corrected 95% CI = [−0.225, −0.031], *p* = 0.008). Because the confidence interval did not include zero, H3d was supported.

In summary, AI literacy moderated the relationships between job replacement anxiety and the two forms of regulatory focus and also moderated the corresponding indirect effects through regulatory focus. Therefore, H3a, H3b, H3c, and H3d were supported, and H3 was supported. As a robustness check, the integrated structural model was re-estimated after replacing the ordinal-coded control variables with sets of dummy variables. The signs and statistical significance of the focal structural paths, indirect effects, interaction effects, and indices of moderated mediation remained unchanged, indicating that the substantive conclusions were robust to alternative coding of the control variables.

## 5. Discussion

Drawing on conservation of resources theory as the primary explanatory framework, this study constructed and tested a moderated dual-path mediation model to examine how job replacement anxiety influences job crafting from a motivational perspective. Based on three-wave matched survey data, the findings generally supported the proposed theoretical model, while also revealing an inconsistent mediation pattern for approach job crafting. Specifically, this study yielded the following findings.

(1) The relationship between job replacement anxiety and employees’ job crafting showed differentiated patterns, suggesting that job crafting under AI replacement pressure is complex.

The results showed that the total effect of job replacement anxiety on approach job crafting was not significant, whereas job replacement anxiety had a significant positive effect on avoidance job crafting. This suggests that job replacement anxiety does not simply lead to job crafting in a single direction. Instead, employees may adopt qualitatively different responses to AI-related replacement threats.

Specifically, the findings for approach job crafting suggest two competing responses. Job replacement anxiety may weaken employees’ willingness to invest additional resources in growth, exploration, and role expansion. However, the same threat may also stimulate compensatory proactive adaptation. Employees who are concerned about being replaced by AI may seek to preserve employability and enhance irreplaceability by acquiring new skills, assuming broader responsibilities, or proactively adjusting jobs to technological change. These opposing tendencies provide a plausible explanation for the inconsistent mediation pattern. However, due to the limitations of the available data, the present study cannot determine whether this proactive job-preservation explanation, or another mechanism such as professional-identity protection or future-oriented employability concerns, accounts for the competing positive process. In contrast, the positive relationship with avoidance job crafting is consistent with efforts to limit further losses by reducing resource expenditure, avoiding uncertain tasks, or narrowing role boundaries.

With regard to avoidance job crafting, the career security threat and expectations of resource loss triggered by job replacement anxiety are more likely to activate employees’ need for resource protection. Employees may therefore seek to reduce potential losses by lowering resource consumption, avoiding uncertain tasks, and narrowing role boundaries, which explains the significant positive effect on avoidance job crafting.

These findings also echo existing research identifying job crafting as an important response to AI-driven change (Cai & Yu, 2024; Gui et al., 2024; He et al., 2024; Mo et al., 2024; X. Zhang & Zhao, 2026), as well as studies documenting the double-edged effects of AI anxiety (L. Ma & Li, 2025; Y. Zhang & Chen, 2024). By focusing specifically on job replacement anxiety, the present study shows that a direct employment-related threat can be associated with qualitatively different forms of work adjustment rather than a uniform behavioral response.

(2) Promotion focus and prevention focus jointly explain the psychological transformation process linking job replacement anxiety to different types of job crafting.

The results showed that job replacement anxiety was negatively associated with promotion focus, which was positively associated with approach job crafting. At the same time, job replacement anxiety was positively associated with prevention focus, which was positively associated with avoidance job crafting. These findings indicate that regulatory focus represents an important motivational process through which employees translate AI-related replacement threats into different forms of work adjustment.

The negative promotion-focus pathway is consistent with prior research showing that promotion focus generally facilitates proactive behavior, growth-oriented behavior, and job crafting (Cai & Zhou, 2022; Du & Liu, 2021; J. Wang et al., 2020; Y. Wang & Zhu, 2021). Extending this literature to AI replacement pressure, this study shows how replacement anxiety is associated with growth-oriented motivation and approach-oriented work adjustment. Notably, the promotion-focus pathway captures only one part of the psychological process linking job replacement anxiety to approach job crafting. The inconsistent mediation pattern indicates that, although the proposed promotion-focus mechanism is theoretically meaningful, it does not provide a complete account of employees’ responses to replacement threats. Thus, promotion focus should be understood as an important but nonexclusive motivational pathway linking job replacement anxiety to approach job crafting.

The positive prevention-focus pathway is consistent with prior research suggesting that stressful or demanding situations tend to activate prevention focus and further induce avoidant or defensive behaviors (Jiao et al., 2024; J. Wang et al., 2020). It is also consistent with studies showing that approach-oriented and avoidance-oriented job crafting follow different logics and lead to different outcomes (Hou et al., 2022; H. Li et al., 2022; Miao & Du, 2025). Compared with the more complex approach pathway, this pattern suggests a more direct motivational route from replacement threat to vigilance and avoidance-oriented work adjustment.

(3) AI literacy buffers the transformation of job replacement anxiety into the two types of regulatory focus and further weakens the corresponding indirect effects.

The results showed that AI literacy played an important boundary role in the process through which job replacement anxiety influenced employees’ job crafting. On the one hand, AI literacy weakened the negative effect of job replacement anxiety on promotion focus and further reduced the negative indirect effect of job replacement anxiety on approach job crafting through promotion focus. On the other hand, AI literacy also weakened the positive effect of job replacement anxiety on prevention focus and further reduced the positive indirect effect of job replacement anxiety on avoidance job crafting through prevention focus.

These findings extend prior research showing that technological literacy serves as an important resource foundation for individuals’ adaptation to digital transformation, and that improving digital skills helps strengthen individuals’ technological adaptability (P. Li et al., 2025; B. Wang et al., 2023). In addition, prior research has shown that AI literacy can increase individuals’ willingness to use AI (Yu et al., 2026) and influence employees’ cognitive and emotional responses when facing AI anxiety (Tan & Wang, 2025). Consistent with these studies, the present study further positions AI literacy as a boundary condition for employees’ motivational responses to replacement pressure. Importantly, although higher AI literacy attenuated the prevention-focus pathway, it did not eliminate it, suggesting that AI literacy functions as a buffer rather than a complete offsetting force.

In summary, the findings suggest that whether employees can achieve sustained adaptation in the AI era depends not only on AI technology itself, but also on how they perceive job replacement threats, how they form self-regulatory orientations, and whether they possess sufficient AI literacy to reappraise technological change. Accordingly, this study connects research on AI literacy, job replacement anxiety, regulatory focus, and job crafting, providing a more nuanced explanation of employees’ psychological mechanisms and behavioral responses during AI-driven organizational transformation.

### 5.1. Theoretical Implications

The theoretical contributions of this study are mainly reflected in the following aspects.

(1) This study further narrows AI anxiety research to job replacement anxiety, a specific dimension that is more directly related to job security. Previous studies have mostly examined the effects of AI anxiety on behavioral outcomes such as employee innovation, knowledge search, technology use, and job crafting at a general level (L. Ma & Li, 2025; Tan & Wang, 2025; Y. Zhang & Chen, 2024). However, AI anxiety encompasses multiple dimensions, including learning anxiety, sociotechnical blindness anxiety, ethical and privacy anxiety, and job replacement anxiety. Treating AI anxiety as a holistic construct may obscure the most direct job security threat that employees experience when facing AI. By taking job replacement anxiety as the entry point, this study reveals how this specific dimension of anxiety influences employees’ subsequent work adaptation behavior. This approach helps move AI anxiety research from general technological anxiety toward more specific explanations grounded in occupational contexts, and makes research on employees’ behavioral responses in AI contexts more closely aligned with changes in the labor market and transformations of organizational roles.
(2) Drawing on conservation of resources theory as the primary explanatory framework, this study extends the understanding of how job crafting emerges in AI contexts. Existing job crafting research has emphasized that employees adjust their work tasks, relationships, and cognitive boundaries through bottom-up processes to achieve better person-job fit (Bruning & Campion, 2018; Tims & Bakker, 2010; Wrzesniewski & Dutton, 2001). In recent years, scholars have also begun to examine the effects of digitalization and AI contexts on job crafting (He et al., 2024; Mo et al., 2024). However, explanations for why AI replacement pressure has differential relationships with different types of job crafting remain relatively limited. More importantly, the findings provide a more differentiated application of conservation of resources theory to AI-related replacement threats. Employees’ responses cannot be fully understood as a uniformly defensive or loss-avoidance response. Although replacement anxiety can weaken growth-oriented motivation and thereby constrain approach-oriented job adjustment, it may simultaneously activate compensatory efforts to preserve employability and occupational value. This coexistence of resource-conservation and proactive adaptation provides a more differentiated application of conservation of resources theory in the context of AI-related replacement threats, suggesting that the same anticipated resource threat may generate competing adaptation strategies. Meanwhile, job replacement anxiety also promotes avoidance job crafting through prevention focus, reflecting a more direct resource-protection response. Therefore, under AI replacement pressure, job crafting may involve both proactive attempts to maintain or rebuild valued resources and defensive efforts to minimize further resource loss. This perspective helps deepen the understanding of how resource threats can generate heterogeneous forms of employee adaptation during AI-driven organizational change.
(3) This study clarifies the boundary condition under which job replacement anxiety influences employees’ job crafting by introducing AI literacy as a moderating variable. Existing research on AI literacy has often emphasized it as a set of competencies related to understanding, using, evaluating, and making ethical judgments about AI technologies, with particular attention to using AI, technology acceptance, or learning adaptation (H. Li & Zhao, 2025; B. Wang et al., 2023). This study further demonstrates that AI literacy is not only a technical competency, but also an important personal resource that shapes employees’ stress appraisal and motivational transformation. AI literacy can change how employees interpret AI replacement threats, thereby influencing the extent to which job replacement anxiety is transformed into promotion focus or prevention focus, and further shaping its indirect effects on job crafting through these two types of regulatory focus. Accordingly, this study extends AI literacy as an important boundary condition for explaining employees’ psychological adaptation and behavioral adjustment in the AI era, providing a new theoretical perspective for understanding employees’ sustained adaptation during intelligent transformation.

### 5.2. Practical Implications

As AI continues to be embedded in organizational production, management, and decision-making processes, organizations should focus not only on the efficiency gains brought by technological applications, but also on employees’ adaptation to job changes, skill renewal, and career development. Based on the findings, this study offers the following three practical implications for organizational management. Given the observational nature of the study, these implications should be interpreted as practice-oriented considerations consistent with the observed associations rather than as evidence that the proposed organizational interventions will causally produce the stated outcomes.

(1) Organizations should pay attention to the management of job replacement anxiety and incorporate employees’ job security into the process of AI transformation. The findings indicate that job replacement anxiety is associated with differences in employees’ job crafting behavior. Therefore, when promoting AI applications, organizations should not only emphasize automation, cost reduction, efficiency improvement, and process optimization, but also help employees clarify the specific implications of AI applications for job tasks, competency requirements, and career development paths. For example, organizations can help employees distinguish between repetitive tasks that may be replaced by AI and core tasks that still require human judgment, communication, creativity, and integrative capabilities through job task mapping, explanations of human–AI collaboration scenarios, and career development communication. By improving the comprehensibility and predictability of job changes, organizations can reduce employees’ excessive threat perceptions arising from uncertainty and create conditions for them to engage in proactive work adjustment behaviors.
(2) Organizations should create a management environment that supports employees’ approach to job crafting and prevents AI transformation from inducing excessive defensiveness and avoidance. The findings suggest that job replacement anxiety is associated with more constrained approach-oriented work adjustment and greater avoidance-oriented responses. One possible contextual explanation for this pattern concerns the cultural norms surrounding employee-initiated work adjustment. In the Chinese context, greater sensitivity to hierarchy and interpersonal harmony may make some employees more cautious about independently altering task or relational boundaries under uncertainty, which could partly explain why job replacement anxiety is associated with weaker approach-oriented adjustment and stronger avoidance-oriented responses. Therefore, when promoting AI adoption, organizations should stimulate employees’ growth orientation and willingness to explore by increasing job autonomy, establishing AI pilot projects, encouraging employee participation in process optimization, and providing room for trial and error as well as positive feedback. For employees who show clear avoidance tendencies and withdrawal behaviors, managers should provide additional resources such as training time, mentoring support, and task reallocation, so as to prevent prolonged anxiety from leading to sustained withdrawal.
(3) Organizations should regard AI literacy development as an important means of enhancing employees’ sustainable adaptability. The findings indicate that AI literacy can buffer the effects of job replacement anxiety on employees’ subsequent psychological states and job crafting behavior. Therefore, AI literacy training should not be limited to tool-use training, but should cover basic AI knowledge, job-specific application scenarios, outcome evaluation, human–AI collaboration methods, and ethical risk identification. Organizations can enhance employees’ AI literacy through systematic training and practice mechanisms tailored to different job roles. For example, they can provide AI skills training, promote cross-departmental digital learning programs, and offer opportunities for employees to practice using AI tools, thereby gradually improving employees’ understanding and application of AI technologies. At the same time, organizations should encourage employees to explore how AI tools can be integrated with actual business scenarios. This can strengthen employees’ confidence and adaptability in technology-driven environments and make them more likely to view AI as a resource for enhancing work capability and career competitiveness, rather than merely as a replacement threat.

### 5.3. Limitations and Future Research

Although this study examined the relationships among job replacement anxiety, regulatory focus, job crafting, and AI literacy in the AI context, several limitations remain. Future research can be further advanced in the following directions.

(1) Although this study adopted a three-wave matched questionnaire design, the data remain observational and were primarily based on self-reports. The three-wave design reduced same-source concerns to some extent, but it does not establish causal ordering or fully rule out common method bias and reverse causality. Accordingly, the relationships identified in this study should be interpreted as associations rather than definitive causal effects. Future research could adopt multi-source data collection methods, such as combining employee self-reports, supervisor evaluations, and objective behavioral data, to further improve the robustness of the findings. In addition, experimental methods, longitudinal designs, or diary studies could be used to capture dynamic changes in employees’ psychological states and job crafting behavior during AI implementation, thereby revealing the temporal process through which job replacement anxiety influences employee behavior in greater detail.
(2) The generalizability of the findings is constrained by the specific sampling and empirical context of this study. Participants were employees working in China and were recruited through referral-based online recruitment and organization-assisted recruitment, both of which are non-probability sampling procedures. Moreover, all questionnaires were completed electronically, which may have increased the likelihood of participation among employees who were more accustomed to digital technologies or more receptive to AI, thereby introducing potential self-selection and coverage bias. The findings therefore should not be assumed to generalize directly to the broader employee population in China or to other national, occupational, industrial, or technological contexts. Employees’ responses to AI may vary with demographic and occupational characteristics, organizational digitalization, access to AI tools, regulatory environments, and the specific form and intensity of AI use. Future research could employ probability-based or stratified sampling, recruit participants across different countries, industries, organizations, occupations, and demographic groups, and conduct cross-group or cross-national comparisons. It would also be useful to distinguish among different AI technologies and usage contexts, such as generative AI, automation systems, and decision-support technologies, to identify the contextual boundary conditions of the proposed relationships.
(3) The theoretical scope of the present study could also be broadened. Conservation of resources theory was adopted as the primary explanatory framework. However, this perspective may not fully capture growth-oriented or opportunity-seeking processes that can emerge when employees respond proactively to technological change. Future research could therefore integrate conservation of resources theory with complementary perspectives such as self-determination theory, which emphasizes autonomy, competence, and autonomous motivation in explaining proactive and growth-oriented behavior. Such an integrated perspective may help explain why AI-related job replacement concerns can be associated with different forms of adaptation. In addition, the present design cannot distinguish among competing explanations for the inconsistent mediation pattern involving job replacement anxiety, promotion focus, and approach job crafting. Future studies could directly examine alternative mechanisms, such as competence-restoration motives, professional identity protection, self-enhancement processes, or other forms of proactive motivation, to clarify the mechanisms underlying this pattern.
(4) Finally, several limitations are associated with data screening, cross-wave matching, and sample retention. The study only retained the final matched and de-identified dataset; records for respondents who were not included in the final sample, criterion-specific exclusion records, and detailed matching logs were not preserved. Consequently, it was not possible to retrospectively conduct a formal attrition analysis comparing retained and non-retained respondents, determine the number of exclusions attributable to each screening criterion, or reconstruct the exact number of matching conflicts. Potential non-random attrition therefore cannot be completely ruled out. In addition, the identification of regular response patterns involved manual judgment rather than a fully prespecified quantitative rule, which limits the reproducibility of this aspect of data screening. Future research should retain de-identified screening and matching audit records, document the disposition of cases at each wave, and adopt prespecified and reproducible data-quality criteria, thereby enabling a more transparent assessment of attrition and screening-related bias.

## 6. Conclusions

The study shows that job replacement anxiety is not associated with a uniform pattern of job crafting but with differentiated forms of work adjustment. For approach job crafting, the negative indirect association through lower promotion focus coexisted with an opposing positive direct association, resulting in a nonsignificant overall association. This pattern suggests that concerns about AI replacement may involve competing tendencies between reduced growth-oriented motivation and proactive adjustment. In contrast, the relationship with avoidance job crafting followed a clearer resource-protection pathway, with job replacement anxiety positively associated with avoidance job crafting through higher prevention focus. AI literacy attenuated both regulatory-focus pathways and weakened their corresponding indirect associations, although higher AI literacy did not completely remove the prevention-focused response to replacement anxiety. Taken together, the findings indicate that employees’ responses to AI-related replacement concerns involve both resource-acquisition and resource-protection processes, and that AI literacy can buffer the motivational consequences associated with perceived replacement threats.

## Figures and Tables

**Table 1:** Descriptive Statistics of the Sample.

Variable	Category	Frequency	Percentage
Gender	Female	294	50.43%
	Male	289	49.57%
Age	25 years or below	166	28.47%
	26–35 years	243	41.68%
	36–45 years	129	22.13%
	46–55 years	42	7.20%
	56 years or above	3	0.51%
Education level	High school or below	37	6.35%
	Junior college	78	13.38%
	Bachelor’s degree	326	55.92%
	Master’s degree	136	23.33%
	Doctoral degree or above	6	1.03%
Work tenure	0–1 year	136	23.33%
	1–3 years	92	15.78%
	3–5 years	79	13.55%
	5–10 years	115	19.73%
	10–20 years	105	18.01%
	More than 20 years	56	9.61%
Position level	Ordinary employee	340	58.32%
	Frontline manager	143	24.53%
	Middle manager	79	13.55%
	Senior manager	21	3.60%
Frequency of AI product or technology use	Never	7	1.20%
	Occasionally (less than once per week)	62	10.63%
	Sometimes (about once per week)	152	26.07%
	Often (several times per week but not every day)	283	48.54%
	Always (almost every day)	79	13.55%

**Table 2:** Fit Results of the Six-Factor Model and Alternative Models.

Model	χ^2^	df	Δχ^2^	Δdf	RMSEA	AIC	TLI	CFI	IFI
Six-factor model (JRA, AIL, PMF, PVF, APJC, AVJC)	3797.687 ***	1798	-	-	0.044	4107.687	0.923	0.927	0.927
Five-factor model (JRA, AIL, PVF, PMF + APJC, AVJC)	4001.053 ***	1803	203.366 ***	5	0.046	4301.053	0.916	0.920	0.920
Four-factor model (JRA, AIL, PMF + APJC, PVF + AVJC)	4202.256 ***	1807	404.569 ***	9	0.048	4494.256	0.908	0.913	0.913
Three-factor model (JRA + AIL, PMF + PVF, APJC + AVJC)	6845.787 ***	1810	3048.100 ***	12	0.069	7131.787	0.808	0.816	0.817
Single-factor model (JRA + AIL + PMF + PVF + APJC + AVJC)	8968.447 ***	1813	5170.760 ***	15	0.082	9248.447	0.727	0.739	0.739

Note: *N* = 583. Δχ^2^ and Δdf represent differences between each alternative model and the six-factor model. JRA = job replacement anxiety; AIL = AI literacy; PMF = promotion focus; PVF = prevention focus; APJC = approach job crafting; AVJC = avoidance job crafting. *** *p* < 0.001.

**Table 3:** Means, Standard Deviations, and Correlations Among Variables.

Variables	M	SD	1	2	3	4	5	6
1. JRA	3.552	0.855	(0.807)					
2. PMF	3.732	0.607	−0.111 **	(0.907)				
3. PVF	3.978	0.551	0.704 **	0.184 **	(0.906)			
4. APJC	3.649	0.789	0.043	0.791 **	0.394 **	(0.933)		
5. AVJC	2.904	0.862	0.437 **	0.271 **	0.527 **	0.279 **	(0.890)	
6. AIL	3.841	0.483	0.176 **	0.384 **	0.476 **	0.562 **	0.318 **	(0.856)

Note: *N* = 583. Values in parentheses are the square roots of AVE. ** *p* < 0.01. JRA = job replacement anxiety; PMF = promotion focus; PVF = prevention focus; APJC = approach job crafting; AVJC = avoidance job crafting; AIL = AI literacy.

**Table 4:** Structural Path Estimates of the Integrated Latent-Variable Model.

Path	B	SE	β	C.R.	*p*	Lower BC 95% CI	Upper BC 95% CI
JRA → PMF	−0.187	0.030	−0.267	−6.255	<0.001	−0.254	−0.120
JRA → PVF	0.444	0.025	0.714	17.501	<0.001	0.394	0.498
AIL → PMF	0.671	0.058	0.549	11.502	<0.001	0.537	0.798
AIL → PVF	0.425	0.036	0.393	11.894	<0.001	0.337	0.513
JRA × AIL → PMF	0.173	0.050	0.135	3.431	<0.001	0.043	0.303
JRA × AIL → PVF	−0.100	0.031	−0.089	−3.207	0.001	−0.184	−0.020
PMF → APJC	1.247	0.072	0.911	17.290	<0.001	1.115	1.401
JRA → APJC	0.162	0.025	0.168	6.461	<0.001	0.106	0.222
PVF → AVJC	1.220	0.135	0.732	9.072	<0.001	0.910	1.577
JRA → AVJC	−0.074	0.074	−0.071	−0.993	0.321	−0.265	0.103

Note: *N* = 583. JRA = job replacement anxiety; AIL = AI literacy; PMF = promotion focus; PVF = prevention focus; APJC = approach job crafting; AVJC = avoidance job crafting.

**Table 5:** Bootstrap Tests of Total and Indirect Effects at the Mean Level of AI Literacy.

Path	Effect	Boot SE	Lower BC 95% CI	Upper BC 95% CI
JRA → APJC (total effect)	−0.072	0.042	−0.150	0.014
JRA → PMF → APJC	−0.234	0.043	−0.317	−0.149
JRA → AVJC (total effect)	0.468	0.053	0.361	0.571
JRA → PVF → AVJC	0.542	0.071	0.413	0.694

Note: *N* = 583. JRA = job replacement anxiety; PMF = promotion focus; PVF = prevention focus; APJC = approach job crafting; AVJC = avoidance job crafting.

**Table 6:** Results of the Simple Slope Analysis.

Path	AI Literacy Level	Effect	Boot SE	Lower BC 95% CI	Upper BC 95% CI
JRA → PMF	Low (M − 1SD)	−0.268	0.054	−0.373	−0.159
	Mean	−0.187	0.035	−0.254	−0.120
	High (M + 1SD)	−0.107	0.037	−0.181	−0.036
JRA → PVF	Low (M − 1SD)	0.490	0.039	0.418	0.570
	Mean	0.444	0.026	0.394	0.498
	High (M + 1SD)	0.397	0.025	0.352	0.449

Note: *N* = 583. JRA = job replacement anxiety; AIL = AI literacy; PMF = promotion focus; PVF = prevention focus.

**Table 7:** Conditional Indirect Effects and Indices of Moderated Mediation.

Path	AI Literacy Level	Effect	Boot SE	Lower BC 95% CI	Upper BC 95% CI
JRA → PMF → APJC	Low (M − 1SD)	−0.334	0.065	−0.459	−0.202
	Mean	−0.234	0.043	−0.317	−0.149
	High (M + 1SD)	−0.134	0.048	−0.230	−0.041
Index of moderated mediation: PMF pathway	—	0.216	0.079	0.056	0.364
JRA → PVF → AVJC	Low (M − 1SD)	0.599	0.080	0.455	0.769
	Mean	0.542	0.071	0.413	0.694
	High (M + 1SD)	0.485	0.070	0.363	0.641
Index of moderated mediation: PVF pathway	—	−0.123	0.049	−0.225	−0.031

Note: *N* = 583. JRA = job replacement anxiety; AIL = AI literacy; PMF = promotion focus; PVF = prevention focus; APJC = approach job crafting; AVJC = avoidance job crafting.

## Data Availability

The data presented in this study are available on reasonable request from the corresponding author. The data are not publicly available due to privacy and ethical restrictions.

## Appendix A

**Table A1:** Measurement Information and Representative Items.

Construct	(Sub-)Dimension/Content Domain		Items	Representative Item	Cronbach’s α	Wave	Response Format
Job replacement anxiety	/		6	I worry that AI technology/products will replace some people’s jobs.	0.917	Time 1	Five-point agreement scale ranging from 1 (strongly disagree) to 5 (strongly agree)
AI literacy	Awareness		3	I can distinguish between devices that are AI-enabled and those that are not.	0.775		
	Use		3	I can proficiently use AI applications or products to assist me in completing my daily work.	0.784		
	Evaluation		3	After using an AI application or product for some time, I can evaluate its capabilities and limitations.	0.806		
	Ethics		3	I always follow ethical principles when using AI applications or products.	0.777		
Regulatory focus	Promotion focus	Gains	3	I take chances at work to maximize my goals for advancement.	0.800	Time 2	
		Achievement	3	Whether my current job provides opportunities for advancement is extremely important for me.	0.837		
		Ideals	3	I spend a great deal of time envisioning how to fulfill my career aspirations.	0.798		
	Prevention focus	Security	3	I focus on completing work tasks correctly to enhance my job security	0.854		
		Oughts	3	At work, I focus on fulfilling my assigned responsibilities.	0.850		
		Losses	3	I do everything I can to avoid losses at work.	0.847		
Job crafting	Approach job crafting	Work-role expansion	5	I provide input on important issues to enrich my work role.	0.926	Time 3	Five-point frequency scale ranging from 1 (almost never) to 5 (always).
		Technology adoption	5	I use new knowledge or technologies to automate my work and improve work efficiency.	0.933		
		Social expansion	4	I actively develop my professional network at work.	0.920		
		Metacognition	5	I find ways to prepare myself for future work.	0.918		
	Avoidance job crafting	Work-role reduction	4	I work in ways that allow me to avoid tedious tasks at work.	0.895		
		Withdrawal	3	I find ways to reduce the amount of time I spend in meetings.	0.814		

**Table A2:** Heterotrait–Monotrait Ratios Among the Focal Constructs.

Construct	1	2	3	4	5	6
1. JRA	-					
2. PMF	0.125	-				
3. PVF	0.766	0.213	-			
4. APJC	0.056	0.847	0.420	-		
5. AVJC	0.480	0.294	0.579	0.298	-	
6. AIL	0.195	0.434	0.523	0.605	0.355	-
